# Supplementary material for: Multiscale computational evaluation of Vitex trifolia phytochemicals as VEGFR2 inhibitors for targeted breast cancer therapy
Source: PLoS One. 2025 Jun 10;20(6):e0325255. doi: 10.1371/journal.pone.0325255 (PMC12151475; doi:10.1371/journal.pone.0325255)
Supplement: S1 File — (DOCX) [file pone.0325255.s001.docx]

**Supplementary data**


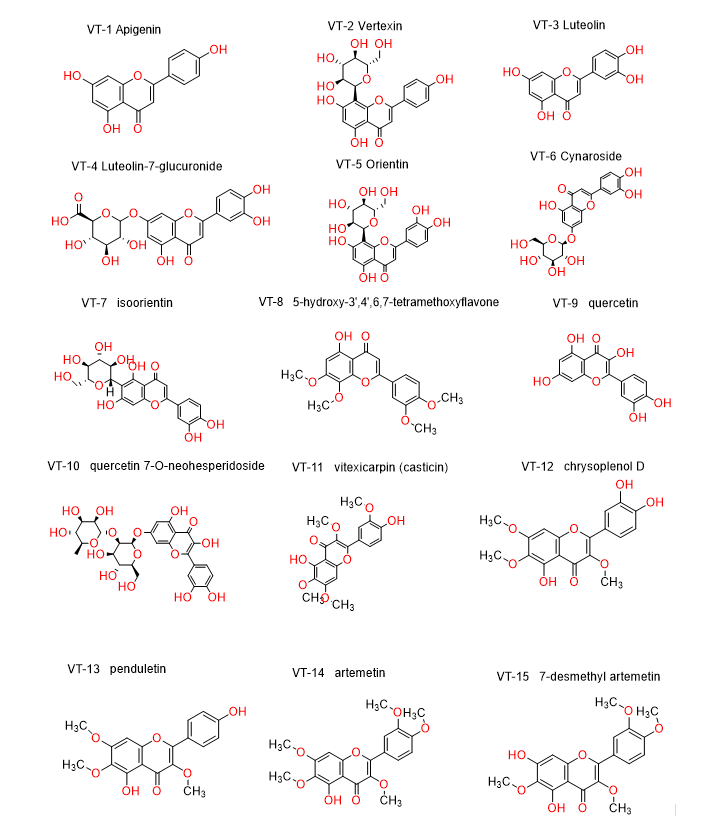


**Figure S1: Chemical structures of Phytochemicals of *Vitex trifolia* used in the In silico analysis to predict a potential inhibitor against BC**

**Table S1: 2D, 3D Structures, and SMILES of Phytochemicals of *Vitex trifolia* used in the *In Silico* analysis to predict a potential inhibitor against BC**

| **Code** | **Comp. Name** | **2D Structure** | **3 D Structure** | **SMILES** |
| --- | --- | --- | --- | --- |
| **VT-1** | **apigenin** |  | 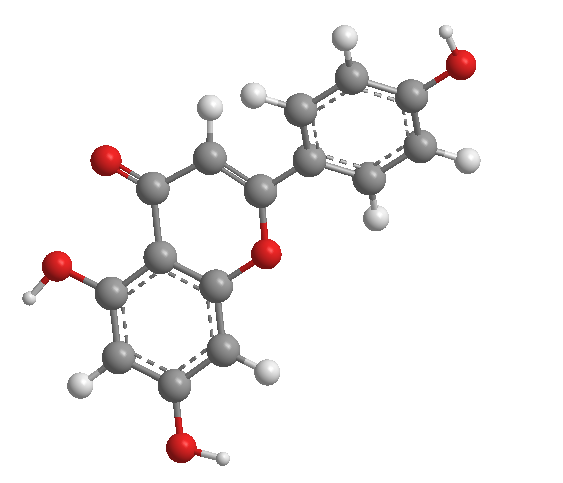 | O=C1C=C(C2=CC=C(O)C=C2)OC3=C1C(O)=CC(O)=C3 |
| **VT-2** | **vertexin** |  | 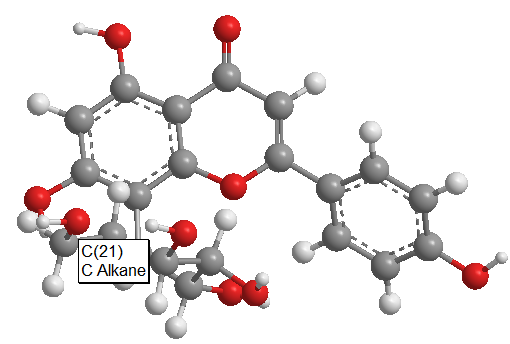 | O=C1C2=C(O)C=C(O)C([C@@H]3O[C@@H](CO)[C@H](O)[C@@H](O)[C@@H]3O)=C2OC(C4=CC=C(O)C=C4)=C1 |
| **VT-3** | **luteolin** |  | 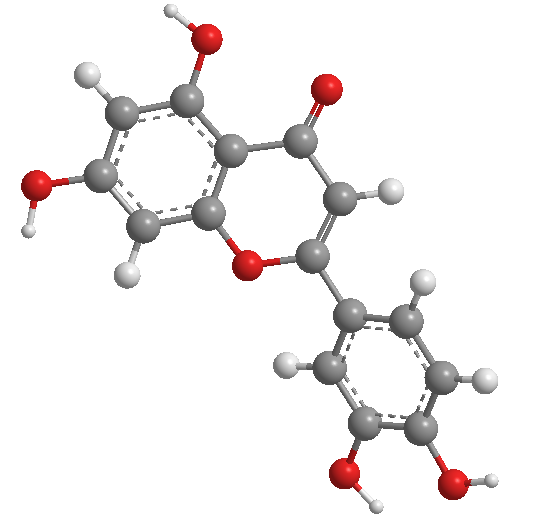 | O=C1C=C(C2=CC=C(O)C=C2)OC3=C1C(O)=CC(O)=C3 |
| **VT-4** | **luteolin-7-glucuronide** |  | 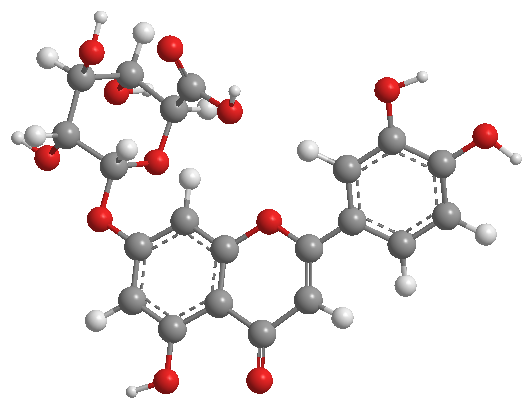 | O=C1C=C(C2=CC=C(O)C(O)=C2)OC3=C1C(O)=CC(OC4[C@@H]([C@H]([C@@H]([C@@H](C(O)=O)O4)O)O)O)=C3 |
| **VT-5** | **orientin** |  | 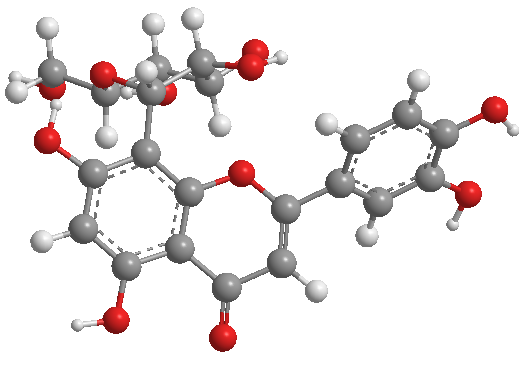 | O=C1C2=C(O)C=C(O)C([C@@H]3O[C@@H](CO)[C@H](O)[C@@H](O)[C@@H]3O)=C2OC(C4=CC=C(O)C(O)=C4)=C1 |
| **VT-6** | **cynaroside** |  | 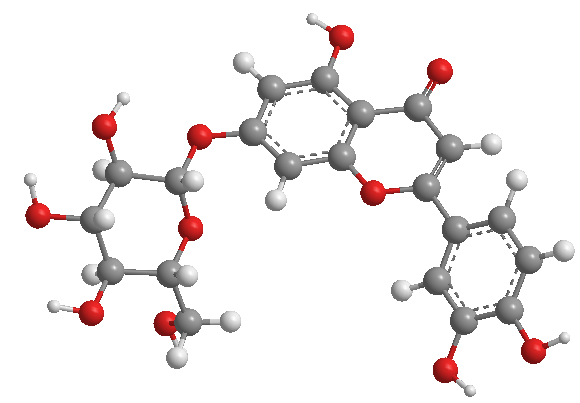 | O[C@@H]1[C@@H](CO)O[C@@H](OC2=CC(OC(C3=CC(O)=C(O)C=C3)=CC4=O)=C4C(O)=C2)[C@H](O)[C@H]1O |
| **VT-7** | **isoorientin** |  | 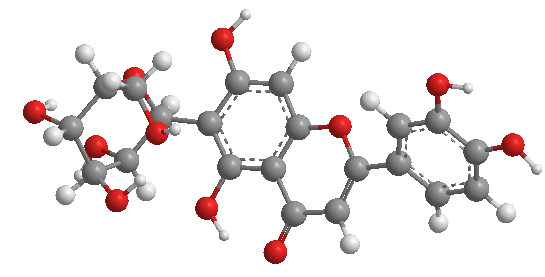 | OC1=C([C@@]2([H])[C@H](O)[C@@H](O)[C@H](O)[C@@H](CO)O2)C(O)=CC(O3)=C1C(C=C3C4=CC(O)=C(O)C=C4)=O |
| **VT-8** | **5-hydroxy-3',4',6,7-tetramethoxyflavone** |  | 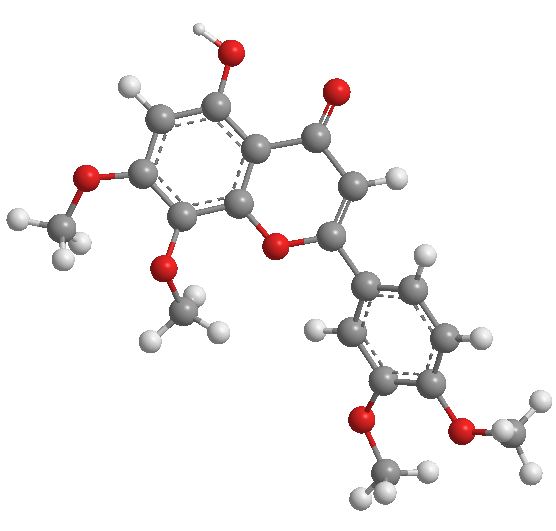 | COC1=C(OC)C=CC(C2=CC(C(C(O)=CC(OC)=C3OC)=C3O2)=O)=C1 |
| **VT-9** | **quercetin** |  | 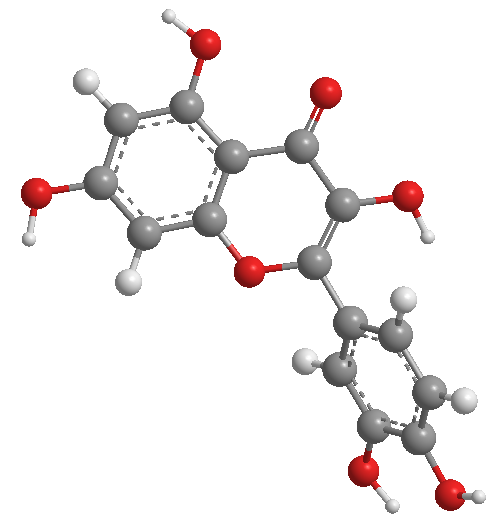 | OC1=C(O)C=C(C2=C(O)C(C(C(O)=CC(O)=C3)=C3O2)=O)C=C1 |
| **VT-10** | **quercetin 7-*O*-neohesperidoside** |  | 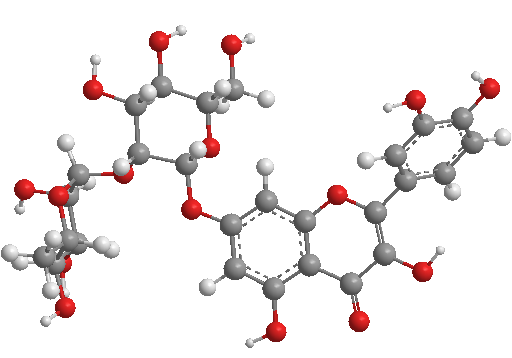 | OC1=CC=C(C2=C(O)C(C3=C(O)C=C(O[C@H]4O[C@@H](CO)[C@H](O)[C@@H](O)[C@H]4O[C@H]5[C@H](O)[C@H](O)[C@@H](O)[C@H](C)O5)C=C3O2)=O)C=C1O |
| **VT-11** | **vitexicarpin (casticin)** |  | 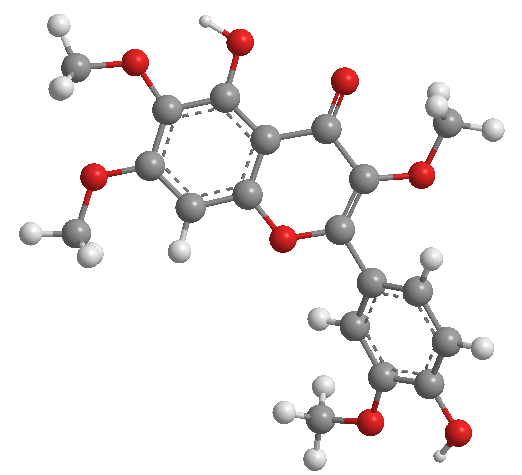 | OC1=C(OC)C=C(C2=C(OC)C(C(C(O)=C(OC)C(OC)=C3)=C3O2)=O)C=C1 |
| VT-12 | **chrysoplenol D** |  | 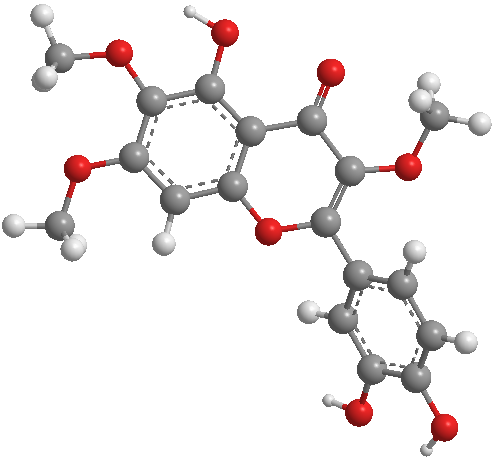 |  |
| VT-13 | **penduletin** |  | 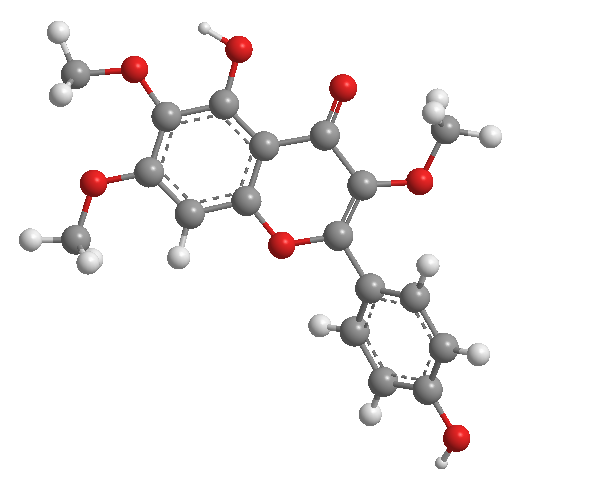 |  |
| **VT-14** | **artemetin** |  | 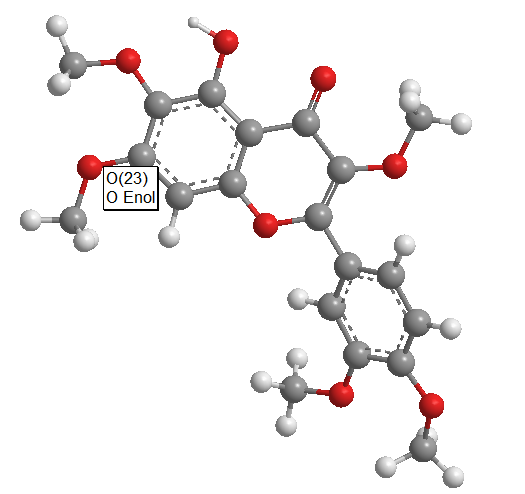 | O=C1C2=C(C=C(OC)C(OC)=C2O)OC(C3=CC(OC)=C(OC)C=C3)=C1OC |
| **VT-15** | **7-desmethyl artemetin** |  | 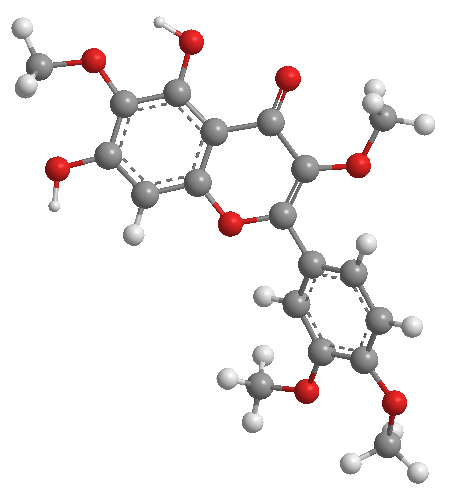 | O=C1C2=C(C=C(O)C(OC)=C2O)OC(C3=CC(OC)=C(OC)C=C3)=C1OC |
| **CCL** | Axitinib |  | 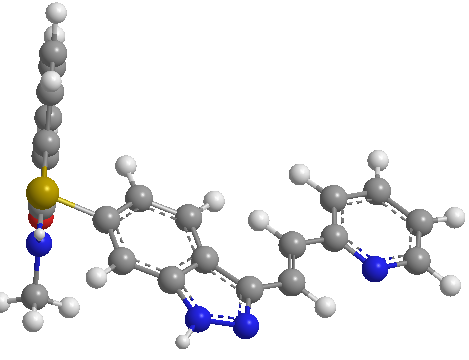 | CNC(=O)C1=CC=CC=C1SC2=CC3=C(C=C2)C(=NN3)/C=C/C4=CC=CC=N4 |

**Table S2 :** Hydrophobic interactions showing bond distances between compounds and protein residues

| **Compound** | **Residue** | **Amino Acid** | **Distance (Å)** |
| --- | --- | --- | --- |
| CCL | 840 | LEU | 3.73 |
|  | 848 | VAL | 3.59 |
|  | 866 | ALA | 3.93 |
|  | 889 | LEU | 3.56 |
|  | 899 | VAL | 3.27 |
|  | 916 | VAL | 3.55 |
|  | 918 | PHE | 3.09 |
|  | 1035 | LEU | 3.10 |
|  | 1044 | ILE | 3.63 |
| VT-1 | 848 | VAL | 3.33 |
|  | 866 | ALA | 3.73 |
|  | 885 | GLU | 3.94 |
|  | 889 | LEU | 3.60 / 3.96 |
|  | 899 | VAL | 3.91 |
|  | 1046 | ASP | 3.59 |
| VT-2 | 840 | LEU | 3.68 |
|  | 918 | PHE | 3.91 |
| VT-3 | 848 | VAL | 3.42 |
|  | 866 | ALA | 3.98 |
|  | 889 | LEU | 3.51 |
|  | 899 | VAL | 3.96 |
|  | 1046 | ASP | 3.55 |
| VT-4 | 848 | VAL | 3.82 |
|  | 866 | ALA | 3.23 |
|  | 889 | LEU | 3.49 |
|  | 899 | VAL | 3.66 |
|  | 1035 | LEU | 3.70 / 3.71 |
|  | 1047 | PHE | 3.66 |
| VT-5 | 918 | PHE | 3.45 / 3.51 |
| VT-6 | 848 | VAL | 3.26 |
|  | 866 | ALA | 3.96 |
|  | 885 | GLU | 3.97 |
|  | 889 | LEU | 3.57 |
|  | 899 | VAL | 3.84 |
|  | 1046 | ASP | 3.76 |
| VT-7 | 848 | VAL | 3.89 |
|  | 885 | GLU | 3.42 |
|  | 899 | VAL | 3.36 |
|  | 916 | VAL | 3.90 |
| VT-8 | 848 | VAL | 3.27 |
|  | 866 | ALA | 3.47 |
|  | 885 | GLU | 3.86 |
|  | 889 | LEU | 3.58 / 3.98 |
|  | 899 | VAL | 3.90 |
|  | 1046 | ASP | 3.62 |
| VT-9 | 848 | VAL | 3.40 |
|  | 866 | ALA | 3.90 |
|  | 885 | GLU | 3.74 |
|  | 889 | LEU | 3.63 |
|  | 899 | VAL | 3.79 |
|  | 916 | VAL | 3.66 |
| VT-10 | 848 | VAL | 3.87 |
|  | 866 | ALA | 3.36 |
|  | 888 | ILE | 3.32 |
|  | 889 | LEU | 3.33 |
|  | 899 | VAL | 3.58 |
|  | 1035 | LEU | 3.69 / 3.85 |
|  | 1047 | PHE | 3.51 |
| VT-11 | 840 | LEU | 3.77 |
|  | 918 | PHE | 3.09 |
| VT-14 | 840 | LEU | 3.76 |
|  | 918 | PHE | 3.11 |
| VT-15 | 885 | GLU | 3.92 |
|  | 888 | ILE | 3.61 |
|  | 889 | LEU | 3.52 |
| VT-16 | 885 | GLU | 3.78 |
|  | 889 | LEU | 3.42 |
|  | 899 | VAL | 3.32 |
| VT-17 | 840 | LEU | 3.50 |

**Table S3. Comparative Binding Free Energy Decomposition Analysis for CCL and VT-6 Complexes.**

| **Complex** | **CCL** | **VT6** |
| --- | --- | --- |
| Δ*E_vdW_ ^a^* | -55.6649 | -54.6919 |
| Δ*E*_ele_ *^a^* | -45.8789 | -34.8113 |
| Δ*G*_nonpol, sol_ *^a^* | -7.6394 | -6.7914 |
| Δggas | -101.5438 | -89.5032 |
| ΔG_sol_ | 51.4792 | 23.5567 |
| Δ*G*_ele, sol (PB)_ *^a^* | 82.1834 | 46.4508 |
| Δ*G*_ele, sol (GB)_ *^a^* | 59.1186 | 30.3482 |
| Δ*E_vdW_*+Δ*G*_nonpol,sol_ *^a^* | -63.3043 | -61.4833 |
| Δ*E*_ele_+Δ*G*_ele,sol (PB)_ *^a^* | 36.3045 | 11.6395 |
| Δ*E*_ele_+Δ*G*_ele,sol (GB)_ *^a^* | 13.2397 | -4.4631 |
| Δ*G*_pred (PB)_*^b^* | -23.5322 | -46.8824 |
| Δ*G*_pred (GB)_*^b^* | -50.0646 | -65.9465 |


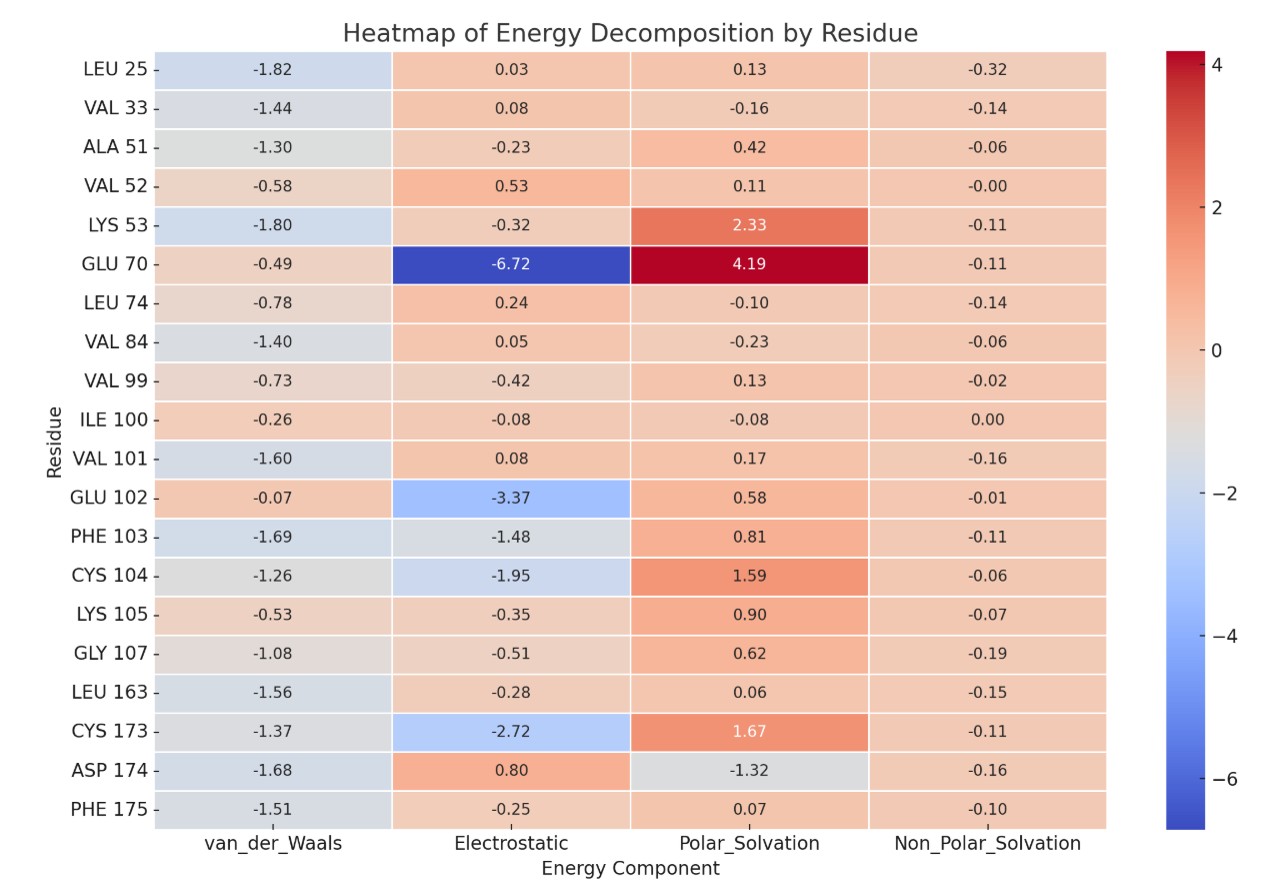


**Figure S2:** Heatmap showing total interaction energy contribution by residue in VT-6


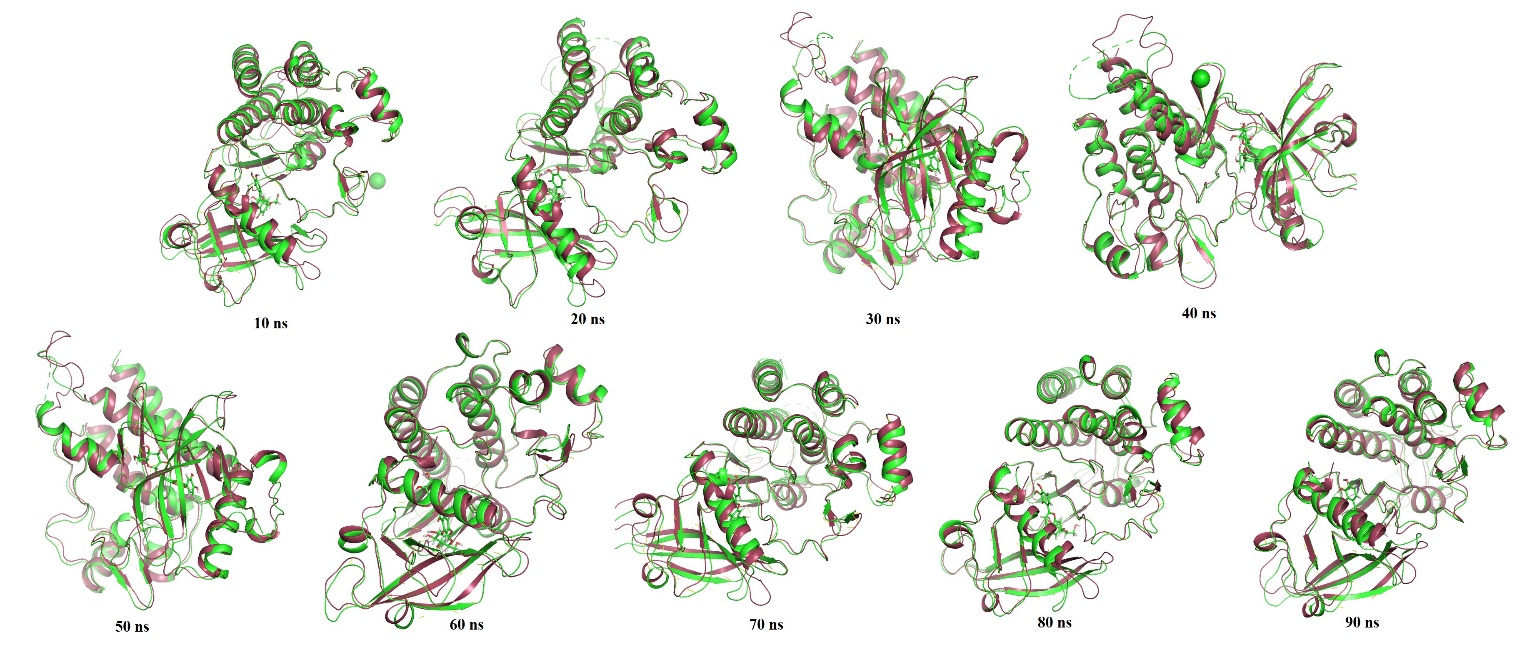


**Figure S3:** Superimposition analysis between the **apo (purple)** form of the protein and CCL (green) complex structures extracted at different nanosecond intervals from the molecular dynamics (MD) simulation.


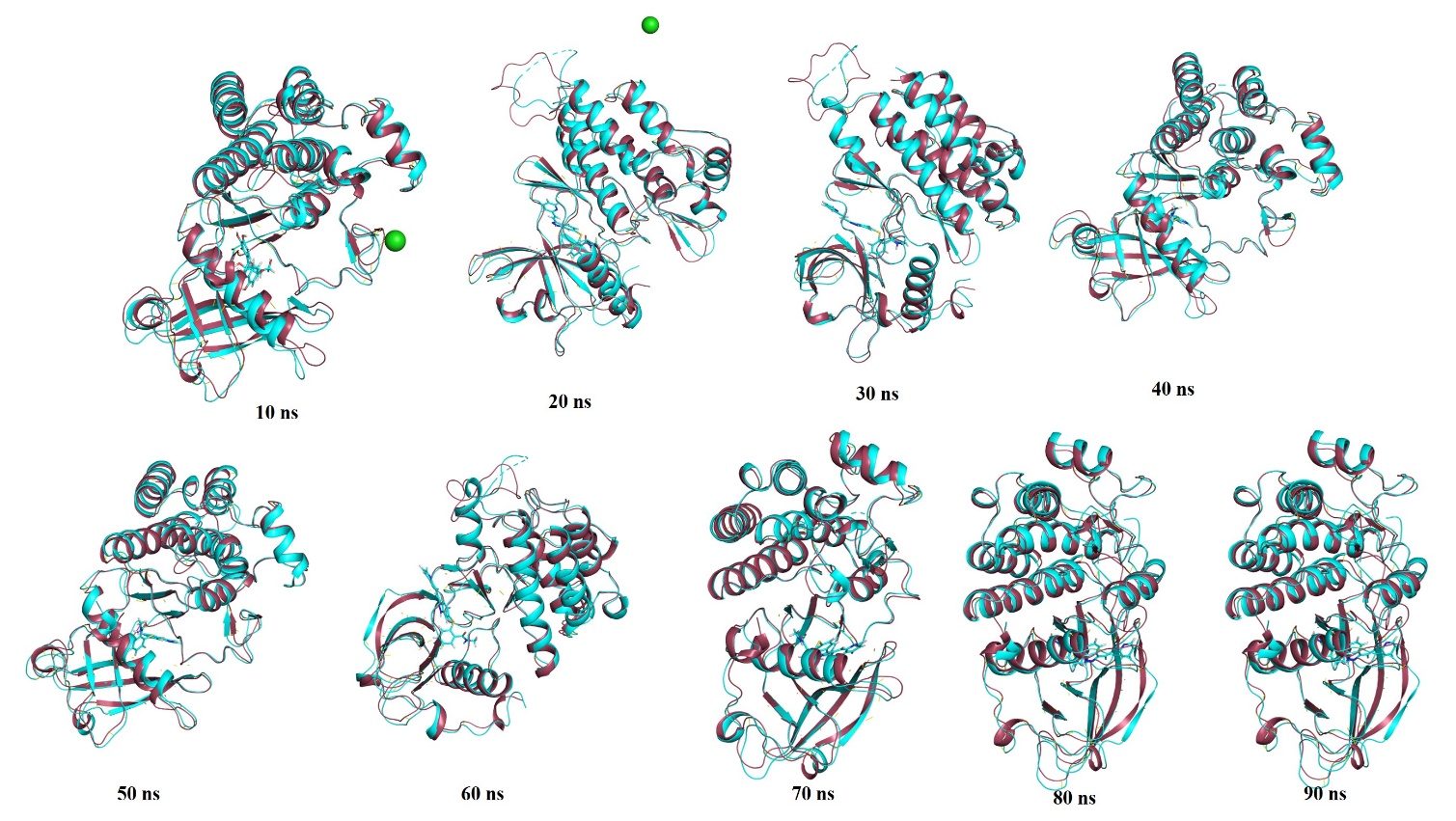


**Figure S4**: Superimposition analysis between the **apo (purple)** form of the protein and VT-6 (cyan) complex structures extracted at different nanosecond intervals from the molecular dynamics (MD) simulation.
